# An exceptional case of myelodysplastic syndrome with myelofibrosis following combination chemotherapy for squamous cell lung cancer

**DOI:** 10.7497/j.issn.2095-3941.2013.02.010

**Published:** 2013-06

**Authors:** Yi-Hao Wang, Rong Fu, Zong-Hong Shao

**Affiliations:** Department of Hematology, Tianjin Medical University General Hospital, Tianjin 300052, China

**Keywords:** DNA methylation, myelodysplastic syndrome, myelofibrosis, squamous cell lung cancer

## Abstract

A 60-year-old woman with squamous cell carcinoma in the right lung was successfully treated with four cycles of combination chemotherapy after surgery, and complete remission was achieved. However, the patient developed myelodysplastic syndrome (MDS) RAEB-2 with myelofibrosis after remission, possibly because of chemotherapy or DNA methylation. The patient responded well to dacitabine (Dacogen), suggesting that DNA hypomethylation agents can be a promising therapy to retard the progression of a second tumor or carcinoma.

## Introduction

Secondary hematological malignancies including myelodysplastic syndrome (MDS) after lung cancer have been reported in previous cases^1^^-^^5^. But secondary MDS with overt myelofibrosis is a rare complication. The estimated relative risk of transforming to hematological malignancy in patients with lung cancer is 37.8 times greater than the age-matched normal population. Possible etiologies include chemotherapy (especially regimens with topoisomerase inhibitor or alkylating agents) and/or irradiation therapy^1^^-^^5^. Hypermethylation of tumor suppressor genes plays an important role in the occurrence of MDS^6^^,^^7^, whether or not hypermethylation is also involved in the pathogenesis of secondary MDS is not well understood. This paper reports a case of MDS with overt myelofibrosis following the combination therapy for lung cancer, in which good efficacy was achieved after the treatment with DNA methyltransferase inhibitor.

## Case report

In March 2007, a 60-year-old female peasant presented with symptoms of persistent cough, with occasional blood-tinged sputum, shortness of breath, and right-side bosom unwell. Chest X-ray showed a mass in the right lung, and computed tomography (CT) scan indicated a nodule shadow approximately 4 cm long in the apical segment of the right lower lobe. Enlarged right hilar lymph nodes or any other evidence of metastatic disease were not found elsewhere. Complete blood cell count was normal, and no hepatosplenomegaly was found. Right, middle, and lower lobectomy, as well as lymph node dissection, were performed. The pathological diagnosis was squamous cell carcinoma. Following four cycles of combination chemotherapy with cisplatin (60 mg/m^2^, IV, day 1), cyclophosphamide (600 mg/m^2^, IV, day 1), and adriamycin (40 mg/m^2^, IV, day 1) after surgery, complete remission was achieved. The patient did not undergo any EGFR-TKI or radiotherapy after surgery. No evidence of recurrence was observed, and no further therapy was undertaken in the three years after the last treatment.

**Figure 1:**
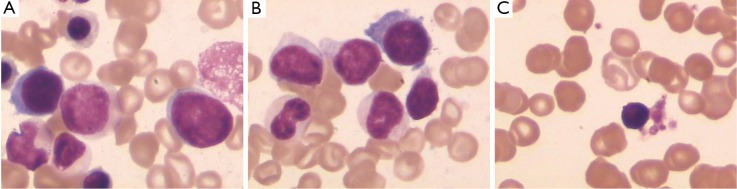
Bone marrow aspirate in the patient with MDS showing myeloblasts (A), auer rods (B), and micromegakaryocyte (C) (Wright-Giemsa, ×1,000).

**Figure 2:**
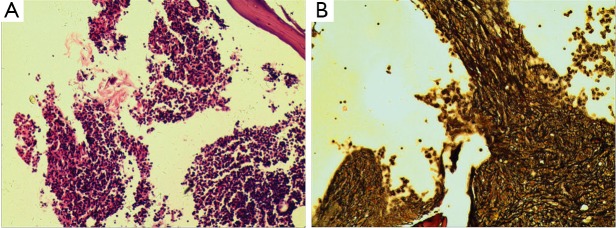
Bone marrow biopsy of the patient with MDS showed hypercellular characteristic (A) (H&E, ×100). Positive (+++) reticular fiber staining indicated myelofibrosis (B) (GMS, ×100).

In April 2010, the patient was again referred to our department because of complaints of dizziness, fatigue, intermittent vomiting, phlegmy cough, and shortness of breath. The blood count showed severe anemia (Hb 49 g/L), leukocytosis (WBC 21×10^9^/L, with a differential of 2% blast cells), and a platelet count of 22×10^9^/L. Peripheral blood smears demonstrated erythrocytic anisocytosis, teardrop poikilocytosis, and immature cells (11% erythroblasts, 2% myeloblasts, 1% promyelocytes). Bone marrow aspirate cytology, cytochemistry, and cytogenic studies were conducted. The bone marrow was hypercellular, with 11.5% myeloblasts. Auer rods and megakaryocytic dysplasia, such as lymphoid megakaryocyte and mononuclear micromegakaryocytes, were observed (**Figure 1**). Both neutrophil alkaline phosphatase and periodic acid-Schiff of the erythroblasts stain were negative. Bone marrow biopsy showed a hypercellular marrow with hemopoietic dysplasia and numerous dysplastic megakaryocytes, indicating MDS. Myeloperoxidase cytochemical staining was strongly positive, with a decreased expression of ki-67. Reticular fiber staining was positive (+++) and indicative of myelofibrosis (**Figure 2**). The immunophenotype of the bone marrow mononuclear showed an R5 gate of 12.4% and also expressed CD34^+^ (47.0%), CD64^+^ (36.6%), CD117^+^ (61.4%), CD15^+^ (39.6%), CD13^+^ (66.5%), CD33^+^ (74.0%), CD11b^+^ (30.4%), CD7^+^ (66.7%), and HLA-DR^+^ (66.4%).

Cytogenetic analysis indicated a normal karyotype (46, XX [9]). The fusion gene of JAK2V617F was positive, and ultrasound scan indicated splenomegaly. Chest CT scan demonstrated normal changes following the resection of the right lung carcinoma without any special abnormality, such as new nodules. The serum tumor markers of lung carcinoma were all negative. The clinical diagnosis was MDS RAEB-2 with myelofibrosis, with an IPSS prognostic score of 2 (Intermediate-2).

One cycle of dacitabine (Dacogen) (20 mg/m^2^, days 1 to 5) was immediately adminstrered. Blood count and bone narrow aspirates indicated partial remission. However, the patient refused any further treatment and was discharged against advice. Seven months after discharge, the patient died from unknown causes.

## Discussion

MDS following chemotherapy and/or radiation therapy for lung cancer was observed in previous reports^1^^-^^5^. Complications of MDS have a significantly negative effect on the possibility of long-term survival after lung cancer treatment. MDS subtypes varied from refractory anemia to refractory anemia with excess RAEB-2. However, for patients with this type of MDS, myelofibrosis is a rare complication. In the present case, overt myelofibrosis was diagnosed, and it was based on the positive reticular fiber staining found during bone marrow biopsy, the fusion gene of JAK2V617F, and splenomegaly.

The exact causes of MDS following lung cancer are still unknown. In our opinion, the possible causes of this type of MDS are chemotherapy, radiation therapy, and/or the accumulation of DNA methylation. In conventional lung cancer treatment, chemotherapy and/or radiation therapy are required, especially for small cell lung cancer. Therapy-related MDS following chemotherapy and/or radiation therapy has been reported in lung cancer patients and has been named secondary MDS. Twenty-four percent of lung cancer patients experience secondary MDS, which is closely correlated with chemotherapy regimens and total chemotherapy and/or radiation therapy periods.

Previous studies have indicated that chemotherapy regimens comprising alkylating agents, such as cyclophosphamide and lomustine, prolonged administration of chemotherapy, or additional chemotherapy cycles may be high risk factors of secondary MDS. Our patient received four cycles of combination chemotherapy involving cyclophosphamides for six months. Therefore, the sharp increase in the risk of secondary MDS was realistic. The median latent period from the start of lung cancer chemotherapy to MDS development varies from 10 to 43 months. In this case, the MDS diagnosis was made 37 months after lung cancer remission. These results support the causal relationship between the use of combination chemotherapy and MDS complications. Moreover, these findings also suggest that new and effective treatments should be introduced to reduce or replace alkylating agents in chemotherapy regimens. The risk of secondary MDS in patients with lung cancer may then be eliminated or lowered to an acceptable level.

Patients with secondary MDS typically have clonal cytogenetic abnormalities, such as a partial or complete lack of either chromosome no. 5 or chromosome no. 7. However, the patient in this case report had normal chromosomes. In addition, secondary MDS can progress more rapidly and is more difficult to treat than *de novo* MDS. The median lifetime of patients diagnosed with secondary MDS after lung cancer is less than six months, but this patient managed to survive for eight months after she was diagnosed with MDS. Therefore, this case does not have the features of secondary MDS and is possibly a case of *de novo* MDS as a result of accumulation of DNA methylation, which is supported by the positive response to the DNA methyltransferase inhibitor Dacogen. DNA methylation is an important regulator of gene transcription. Hypermethylation occurs in a tumor-specific pattern in CpG island sequences at the promoter regions of tumor suppressor genes and other cancer-related genes, as well as represses transcription. As a result, stable gene silencing occurs, which plays a critical role in tumorigenesis. Hypermethylation has been found in virtually all types of malignant neoplasms, including MDS and lung cancer. DNA methyltransferase inhibitors can inhibit the enzyme responsible for DNA methylation and thus lead to DNA hypomethylation. Hypermethylation-induced gene silencing is then reverted to reactivate tumor suppressor gene transcription, which result in proliferation control and apoptosis sensitivity of tumor cells. As a hypomethylating agent, Dacogen has been approved for use in the treatment of MDS patients by the FDA. In this case, partial remission was obtained after only one Dacogen cycle. The patient may have achieved complete remission and survived longer if she underwent additional cycles of Dacogen treatment. DNA hypermethylation of the suppressor genes can contribute to the pathogenesis of MDS^6^^,^^7^. Similarly, DNA hypermethylation of some genes, such as Wnt antagonist SFRP5, the TIF1γ promoter region, and EGFR, is also critical in the progression of lung cancer^8^^-^^12^. Conventional treatments with surgery and chemotherapy or radiation therapy cannot decrease DNA hypermethylation, and accumulation of DNA methylation results in another tumor or carcinoma, which may be secondary or a co-occurrence with lung cancer. DNA demethyltation treatments reduce the DNA methylation level and thus achieve partial remission and prolonged survival time^13^. The efficacy of DNA methyltransferase inhibitors suggests that hypomethylating agents, such as 5-azacytidine and decitabine, may be introduced to treat lung cancer either alone or in combination with chemotherapy as a new treatment strategy to retard the progression of second tumors or carcinomas, especially MDS.
